# Building the first step: a review of low-intensity interventions for stepped care

**DOI:** 10.1186/1940-0640-7-26

**Published:** 2012-12-11

**Authors:** John McKellar, Julia Austin, Rudolf Moos

**Affiliations:** 1 Center for Healthcare Evaluation, Health Services Research and Development Service, Department of Veterans Affairs Health Care System and Stanford University School of Medicine, Palo Alto, CA, USA; 2 Department of Psychology & Robert Wood Johnson Center for Health Policy, University of New Mexico, Albuquerque, NM, USA

**Keywords:** Alcohol use disorders, Stepped care, Help seeking, Intervention

## Abstract

Within the last 30 years, a substantial number of interventions for alcohol use disorders (AUDs) have received empirical support. Nevertheless, fewer than 25% of individuals with alcohol-related problems access these interventions. If several intensive psychosocial treatments are relatively effective, but most individuals in need do not access them, it seems logical to place a priority on developing more engaging interventions. Accordingly, after briefly describing findings about barriers to help-seeking, we focus on identifying an array of innovative and effective low-intensity intervention strategies, including telephone, computer-based, and Internet-based interventions, that surmount these barriers and are suitable for use within a stepped-care model. We conclude that these interventions attract individuals who would otherwise not seek help, that they can benefit individuals who misuse alcohol and those with more severe AUDs, and that they can facilitate subsequent help-seeking when needed. We note that these types of low-intensity interventions are flexible and can be tailored to address many of the perceived barriers that hinder individuals with alcohol misuse or AUDs from obtaining timely help. We also describe key areas of further research, such as identifying the mechanisms that underlie stepped-care interventions and finding out how to structure these interventions to best initiate a program of stepped care.

## Introduction

Within the last 30 years, a number of psychosocial interventions for alcohol use disorders (AUDs) have received empirical support. Evaluations of these interventions have employed well-controlled randomized trials involving large numbers of individuals and substantial follow-up periods. These trials provide strong support for such interventions as motivational enhancement, cognitive-behavioral treatment, and 12-step facilitation treatment [1]. In addition, it is now clear that individuals who obtain timely help for AUDs have better outcomes than those who do not [2,3].

Despite these advances, only about 25% of individuals with AUDs access any form of help, empirically supported or otherwise [4-7]. When help is sought, it often occurs 10 or more years after the onset of symptoms of disorder [8]. If several intensive psychosocial treatments are relatively effective, but most individuals in need do not access them, it seems logical to place a priority on developing more engaging interventions. Accordingly, after briefly describing findings about barriers to help-seeking, we focus on identifying a palatable array of innovative and effective low-intensity intervention strategies that surmount these barriers and are suitable for use within a stepped-care model.

### Key barriers to help-seeking

Empirical studies of help-seeking over the last two decades highlight a number of reasons why individuals with AUDs delay or never seek treatment. One key set of factors involves individuals’ perceptions of negative concomitants of treatment, including stigma [9,10], dislike of the prevalent group format and the emphasis on spirituality in treatment and self-help groups [10], lack of privacy [10], concern that treatment is ineffective [11], and disinterest in abstinence goals [10,12].

Other common reasons individuals cite for not entering treatment involve a desire for autonomy or a wish to “handle problems more on their own” [9,11,12] and the belief that their alcohol problems are not serious or may improve on their own [10,12,13]. Factors such as the need for childcare [14], the problem of arranging transportation or traveling long distances to care [15,16], and the cost of treatment and lack of adequate insurance coverage also hinder help-seeking [17]. Finally, the time commitment for standard alcohol treatment is high, ranging from nine hours a week for intensive outpatient care to full-time for residential care. Many individuals report a lack of willingness to dedicate such substantial amounts of time to treatment and to accept the resulting interference with responsibilities to family or work [10].

### Stepped-care models

Stepped-care models [18] provide one important method for capitalizing on the appealing qualities of low-intensity interventions, such as their accessibility and autonomy, while providing the opportunity to refer individuals to more intensive treatment when needed. The development of low-intensity initial entrees into treatment is consistent with naturalistic studies of the help-seeking process, which show that individuals often engage in self-quit attempts prior to entering formal or informal treatment [19-21]. Lack of success in a low-intensity intervention could further heighten individuals’ perceptions of the severity of their drinking problems and spur interest in treatment entry, which is readily available within a stepped-care program.

### Low-intensity interventions

A number of low-intensity interventions are suitable for use as a first step of a stepped-care intervention. We focus here on low-intensity interventions that do not require face-to-face interaction. Although there is considerable support for the effectiveness of screening and brief intervention (SBI) in nonspecialty settings such as primary care [22,23], widespread implementation remains elusive. Some of the reasons for low levels of implementation in these settings include lack of training, lack of clinician time, and inadequate reimbursement [24], and the widespread reluctance of providers who are not addiction specialists to talk with patients about alcohol use [25].

We examine three main questions about the suitability of these interventions as candidates for stepped interventions. First, do low-intensity interventions attract individuals who would otherwise not seek help? Second, do individuals who are engaged in heavy alcohol use benefit from these interventions and, importantly, can individuals with moderately or relatively severe AUDs benefit from them? We categorize samples as “moderately to relatively severe” if they include patients diagnosed with alcohol dependence or if their average scores on the Alcohol Use Disorders Identification Test (AUDIT) are >19 and in the range of likely alcohol dependence [26]. Finally, does engagement in a low-intensity intervention inspire subsequent help-seeking when needed? We operationalize “inspiring subsequent help-seeking” as studies where patients seek treatment in the year following the low-intensity intervention. We summarize the evidence presented by these studies, examine their limitations, and discuss issues related to implementation of stepped-care models.

## Methods

### Search procedure

We considered for inclusion peer-reviewed English-language articles that examined alcohol interventions delivered via bibliotherapy, telephone (including Short Message Service [SMS]), computer, or the Internet. Both original articles and meta-analyses were deemed suitable. Our review was performed using the electronic databases of the US National Library of Medicine (PubMed) and the American Psychological Association (PsycInfo) to identify relevant articles published from 1990 through September 2011. The term “alcohol” was combined with the following search terms: web-based intervention, Internet intervention, online intervention, bibliotherapy, text message, book intervention, telephone intervention, remote intervention, computer intervention, and self-help intervention (excluding Alcoholics Anonymous [AA] or 12-step). The term “intervention” was added to most search terms to identify the most relevant articles. In addition to searching electronic databases, we examined potentially relevant articles cited in the identified articles’ reference section [27].

## Results

Using these procedures, we identified a total of 686 articles via PubMed and an additional 382 articles via PsycInfo (1068 unique articles). The titles of all identified articles were examined for correspondence with the study inclusion criteria. This led to the identification of 77 articles of potential interest for further review. The content of these 77 articles was carefully examined by two reviewers who further excluded studies that had the following characteristics: provision of face-to-face care, an insufficient follow-up rate to evaluate the intervention including failure to account for missing data, participant age <18, college samples (which are less representative of clinical samples [28]), or use of highly specialized clinical samples (e.g., a trial targeting pregnant women to decrease incidence of fetal alcohol syndrome). The reviewers identified 18 that met study inclusion and exclusion criteria. These articles are described below in the text and are summarized in Table 1.

**Table 1:** Summary of included studies: salient characteristics and outcomes

**Intervention modality**	**Investigators**	**Study design**	**Sample**	**Elements of intervention**	**Magnitude of alcohol consumption change & dependence measure**	Attract individuals*	Positive outcomes**	**Inspire help-seeking†**
Bibliotherapy	Apodaca & Miller (2003)	Meta-analytic review of 22 bibliotherapy studies	Included studies broadly targeting “problem drinkers”	Included studies invited participants to read and implement self-help materials	Bibliotherapy versus control (self-referred drinkers), d = 0.31	X	XX	X
					Dependence measure: varied across studies (e.g., abstinence rates, alcohol consumption frequency, liver enzyme levels)			
Bibliotherapy	Sobell et al (2002)	Randomized trial	Individuals who consumed >12 drinks per week or ≥5 drinks on ≥5 days in the year prior to assessment	Participants received 1 of 2 written interventions by mail: 1) Motivational enhancement-based feedback and advice (personalized based on participants’ drinking history and patterns); 2) General information on the effects of alcohol, guidelines for safe use, and information on self -monitoring	No significant differences between groups in alcohol consumption change	X	XX	X
		Motivational enhancement/personalized feedback (n = 414);						
		Bibliotherapy/drinking guidelines (n = 411)						
Bibliotherapy	Cunningham et al (2002)	Randomized trial	Individuals who expressed interest in self-help and scored ≥8 on the AUDIT	*Drink Wise*, which uses CBT principles, served as the self-help text. Personalized assessment/feedback was based on the “Drinker’s Check-up” and included personalized assessment, normative feedback, and information regarding the potential negative consequences of alcohol use	Self-help book & personalized assessment/feedback versus single treatment or control, d = 0.21		X	
		Self-help book (n = 22); personalized assessment/feedback (n = 21); self-help book & personalized assessment/feedback (n = 17); no materials (n = 26)						
					Dependence measure: Mean days per week of ≥5 drinks per occasion during a typical week in the 6-month follow-up period			
Bibliotherapy	Bamford et al (2005)	Randomized trial	Individuals presenting for clinic-based alcohol treatment	Participants received a 6-page leaflet based on FRAMES	Leaflet versus no leaflet, d = 0.20		X	X
		Leaflet condition (n = 180); no leaflet condition (n = 181)						
					Dependence measure: Self-rated categorical (yes/no) reduction in alcohol use at follow-up			
Bibliotherapy	Wild et al (2007)	Randomized trial	Current drinkers (used alcohol in the 12 months prior to assessment) with no previous participation in alcohol treatment who had an interest in self-help materials	The self-help pamphlet gave normative feedback regarding drinking habits and included information on the hazards of alcohol use at various consumption levels, guidelines for reducing alcohol use, and referral to a local treatment hotline	Unable to calculate		X	
		Pamphlet (n = 877); no pamphlet (n = 850)						
Bibliotherapy	Kavanagh & Connolly (2009)	Randomized trial	Individuals who met DSM-IV criteria for an AUD	Participants were enrolled in primary care and received information regarding the effects of alcohol, self-monitoring forms, and a self-help booklet. The mailed correspondence treatment further included personalized progress letters and 8 CBT-based newsletters	Dependence measure: Mean drinks per week = 0.39		XX	
		Immediate mailed intervention (n = 103); delayed mailed intervention (n = 101)						
**Telephone-delivered intervention**	Brown et al (2007)	Randomized trial	Individuals who met DSM-IV criteria for alcohol abuse or dependence who were drawn from primary care	The telephone-based intervention consisted of 6 sessions based on MI principles and the stages of change model. Behavioral techniques were used to monitor sobriety goals. Participants were also sent individualized letters summarizing progress after sessions	Telephone-based intervention versus control (male participants) = 0.16		XX	
		Telephone-based intervention (n = 445); control (n = 452)						
					The intervention was not superior to control for women			
					Dependence measure: Total standard drinks consumed in the month prior to 3-month follow-up interview			
Telephone-delivered intervention	Mello et al (2008)	Randomized trial	Noncritically injured emergency department patients who used alcohol at risky levels per NIAAA quantity/frequency guidelines	Targeted individuals with a recent alcohol-related injury. Counselors initiated 2 brief calls (30 minutes & 15 minutes) based on MI principles	No significant differences between groups in alcohol consumption change		X	
		Telephone-based intervention (n = 140); control (n = 145)						
Computer-based intervention	Hester et al (2005)	Randomized trial	Individuals who scored ≥8 on the AUDIT	Participants were given access to computer-based MI. Components include assessment and normative feedback, decisional balance, and negotiating sobriety goals	Pre versus post for computer-based intervention, = 1.05		X	X
		Computer-based intervention (n = 35); control (n = 26)						
					Dependence measure: Mean of 3 dependent variables (average drinks per day, drinks per drinking day, and average peak BAC level) during 12-month follow-up period			
Computer-based intervention	Neumann et al (2006)	Randomized trial	Emergency department patients who scored ≥5 on the AUDIT	Patients in the active treatment condition were given access to computer-delivered personalized advice and normative feedback. Feedback was based on the MI principles and FRAMES guidelines. Other components included information about alcohol and provider referral information	Computer-based intervention versus control = 0.20		X	
		Computer-based intervention (n = 561); control (n = 575)						
					Dependence measure: Proportion of participants who met British Medical Association criteria for at-risk drinking at 6 months post-treatment			
Computer-based intervention	Boon et al (2011)	Randomized trial	Drinkers with alcohol consumption levels exceeding the limits set by the Dutch guidelines for low-risk drinking	Participants in the treatment group received normative feedback and information regarding the negative consequences of alcohol use. Personalized advice was informed by participant drinking patterns, self-efficacy and attitude, and stage of change	Intervention versus control = 0.25		X	
		Computer-based advice (n = 230); control (n = 220)						
					Dependence measure: Meeting or failing to meet Dutch guidelines for low-risk drinking at 1-month follow-up			
Internet-based intervention	Cunningham et al (2009)	Randomized trial	Drinkers who scored ≥4 on the 3 consumption items on the AUDIT-C & expressed an interest in self-help	Participants in the active condition were mailed a URL that allowed them to access the screening program, which provided a personalized assessment and normative feedback	Mailed intervention URL versus control, η_p_^2^ = 0.08		X	
		Sent URL by mail to participate in intervention (n = 92); control (n = 93)						
					Dependence measure: Mean drinks consumed per week during follow-up period			
Internet-based intervention	Pemberton et al (2011)	Quasi-randomized trial	Active duty military personnel	Participants assigned to active treatment received either AS or DCU. Controls received no intervention. Both interventions were adapted for a military population via expert consensus	AS versus control = no significant differences in alcohol consumption change		X	
		Internet-based intervention based on social learning theory (AS) (n = 686);Internet-based intervention based on MI principles (DCU) (n = 1470); control (n = 914)						
					DCU versus control = 0.10			
					Dependence measure: Average drinks per drinking occasion during 1-month follow-up			
Internet-based intervention	Riper et al (2007)	Randomized trial	Drinkers whose consumption levels exceeded Dutch guidelines for low-risk drinking	Online self-help protocol was consistent with CBT and self-control principles. The intervention was accessed via the study website and included goal setting and analysis of alcohol behavior. Participants also had access to a peer-to-peer chat room	Online self-help versus control = 0.40	X	X	
		Online self-help, (n = 130); control (n = 131)						
					Dependence measure: Mean weekly alcohol consumption during 6-month follow-up period			
Internet-based intervention	Postel et al (2010)	Pre-post design	Individuals concerned about their drinking	Therapy was delivered online by a therapist who communicated with the patient asynchronously. Treatment was a blend of CBT and motivational enhancement, along with elements from the stages-of-change model. Therapy assignments were given in 2 stages; patients could choose (with therapist approval) to move to the second stage of treatment	Pre- versus post-intervention = 1.11	X	XX	
		(N = 527)						
					Dependence measure: Mean weekly alcohol consumption immediately post-treatment			
Internet-based intervention	Blankers et al (2011)	Randomized trial	Score >8 on the AUDIT and consumption of >14 standard drinks in a week	SAO: An automated, fully self-guided Internet intervention based on elements of MI and CBT.	TAO versus WL = 0.59 SAO versus WL = 0.36		XX	
		Internet-based self-help, (SAO) (n = 68); Internet-based therapy (TAO) (n = 68); control (WL) (n = 69)						
					Dependence measure: Number of drinks in the 7 days prior to 3-month follow-up			
				TAO: A therapist-led online intervention (elaborated version of SAO’s MI/CBT protocol plus 7 synchronous chat-based therapy sessions)				
				Control: waitlist (WL) condition				
Internet-based intervention	Wallace et al (2011)	Randomized trial	Individuals who accessed the DYD website and scored ≥5 on the AUDIT-C	Participants in the active condition received access to the DYD interactive online alcohol intervention based on CBT, MI, and relapse prevention techniques	DYD versus control = no significant differences in alcohol consumption change		X	
		“Down Your Drink” Internet-based intervention (DYD) n = 3972); control (n = 3963)						
Internet-based intervention (television-supported)	Kramer et al (2009)	Randomized trial	Drinkers whose consumption levels exceeded Dutch guidelines for low-risk drinking	Participants in active treatment were asked to use a CBT-based, television-supported online self-help intervention. The 5-part series depicts a coach guiding 2 individuals with alcohol problems through an intervention. Participants also received written self-help materials and access to the website	Television-based intervention versus control = 0.90		X	
		Television-supported intervention (n = 90); control (n = 91)						
					Dependence measure: Mean weekly alcohol consumption at follow-up			

*Attract Individuals = X indicates the intervention appeared to attract individuals who might otherwise not seek help (defined as those who had not previously sought treatment or who expressed disinterest in formal treatment).

**Positive Outcomes = X indicates the intervention significantly reduced alcohol use; XX indicates the intervention significantly reduced alcohol use in more severe drinkers (alcohol dependence diagnosis or AUDIT >19).

†Inspire help-seeking = X indicates the intervention appeared to be associated with future help-seeking.

Abbreviations: *MI* motivational interviewing, *CBT* cognitive-behavioral therapy, *AUDIT* Alcohol Use Disorders Identification Test, *NIAAA* National Institute on Alcohol Abuse and Alcoholism.

Note: For 4 of the studies, Cohen’s was calculated by the authors using the Effect Size Determination Program (Lipsey & Wilson, 1996) based on either given study chi-square statistics (Bamford et al., 2005; Boon et al., 2011); alcohol consumption outcome summary statistics (Brown et al., 2007); or by comparing the proportion of participants in each condition who no longer met criteria for at-risk drinking as defined by that study (Neumann et al., 2006). Apodaca and Miller (2003) limited effect size calculations to bibliotherapy studies where patients received ≤1 meeting with a clinician. The Cohen’s calculation for Bamford et al. (2005) was based on participants’ reduction in alcohol use. The Cohen’s calculation for Brown et al. (2007) compared alcohol consumption at 3-month follow-up for the experimental group versus control (as opposed to change from baseline). Although the Mello et al. (2008) treatment was not associated with a change in alcohol consumption, impaired driving scores did improve with controls (= 0.31). Pemberton et al. (2011) was designated as quasi-randomized because assignment to active treatment was only available at select study sites. The Cohen’s calculation for Postel et al. (2010) only included participants who completed the treatment (Parts 1 & 2).

### Study effect-size determination

Effect sizes presented (Cohen’s d or η_p_^2^) were obtained directly from the original study calculations in all but four cases [29-32]. Calculations for these four studies were derived from available study statistics using the Effect Size Determination Program from the *Toolkit for Practical Meta-Analysis*[33].

### Bibliotherapy interventions

Bibliotherapy is the provision of written self-help materials to motivate or guide the process of changing drinking behavior. Bibliotherapy may be presented in the form of brief information and education such as in a pamphlet or in the form of a self-guided book or workbook. A meta-analysis by Apodaca and Miller [34] evaluated the effectiveness of a range of bibliotherapeutic interventions. These interventions involved self-guided learning of behavioral and cognitive-behavioral skills aimed at achieving either abstinence or reduction in drinking to nonhazardous levels. Interventions included components such as monitoring alcohol intake, identifying triggers of alcohol use, and setting drinking goals. The vast majority of self-referred participants were recruited via media outlets, and many indicated disinterest in formal treatment options, indicating that the intervention attracted individuals who had not previously sought help.

Compared with control conditions, self-referred individuals who participated in bibliotherapy tended to improve more on problem drinking. Further, there were no significant differences between bibliotherapy and more extensive face-to-face interventions for self-referred individuals, even for interventions that offered up to 12 face-to-face sessions. The authors categorized participants in these studies as problem drinkers without severe dependence, suggesting that severity ranged from alcohol abuse to mild/moderate alcohol dependence. A number of studies noted that individuals who had previously not entered formal treatment or mutual-help groups did so after participating in bibliotherapy. However, it is not possible to establish definitively the effects of bibliotherapy on help-seeking, as the studies did not report subsequent help seeking separately for intervention and control conditions. Studies involving participants who were opportunistically screened (e.g., who were identified by random digit dialing) yielded more heterogeneous outcomes, only some of which tended to support the use of bibliotherapy.

Sobell and colleagues [35] used media outlets (newspapers, television, radio) to recruit participants who had never sought formal treatment and mailed them two versions of an intervention. Participants reported average consumption of >12 drinks per week or consuming five or more drinks on five or more occasions and average AUDIT scores of 20 (low range of alcohol dependence). Individuals in this study were randomized to a self-change condition that included personalized feedback on drinking or to a control condition (receipt of educational materials focused on safe levels of drinking and consequences of harmful drinking). The self-change condition provided personalized feedback describing drinking levels relative to other drinkers, high-risk situations, and motivation to change, and the bibliotherapy version contained information about the effects of alcohol, low-risk drinking guidelines, risky drinking conditions, and drinking logs. No differences were found by intervention type (self-change versus bibliotherapy) at one-year follow-up; individuals in both groups reduced total weekly drinking by an average of 28.3% (p < 0.001) and reduced heavy/binge-drinking days by 33% (p < 0.001). Moreover, almost 25% of participants in both the self-change and the control conditions had sought treatment. Thus, the current study cannot specify that personalized feedback inspired subsequent help-seeking, but the provision of bibliotherapy and repeated assessments present in both conditions may have led participants to seek help in the subsequent year after entering the study.

Cunningham and colleagues [36] assessed the effectiveness of a self-help book and a personalized assessment-feedback intervention both separately and in combination with each other in a general population study. Individuals with AUDIT scores of ≥8 (86 in total) were recruited via use of random-digit dialing and then randomized into conditions including no treatment, self-help book, personalized feedback, or both self-help book and personalized feedback. The self-help book called *Drink Wise: How to Quit Drinking or Cut Down*[37] was chosen based on demonstrated success in prior evaluations [38]. At six-month follow-up, participants randomized to the book and feedback condition achieved better drinking outcomes compared with those randomized to just one of the interventions or to no treatment at all. Specifically, interaction analyses comparing those in the combined group to those in the single-intervention groups report significantly fewer drinks per week (F = 5.4, 1/75 df, p < 0.03; effect size, 0.07) and days per week of five or more drinks per drinking day (F = 19.6, 1/75 df, p < 0.001; effect size, 0.21). This study is one of the few to find a synergistic effect of using feedback in conjunction with self-help materials.

A study by Bamford and colleagues [29] examined the effect of a six-page preparatory leaflet mailed to participants (N = 361) prior to entering treatment. The study randomized individuals scheduled to enter specialty alcohol treatment to an intervention condition that received the preparatory pamphlet or to a no-pamphlet control group. The preparatory leaflet was based on the FRAMES acronym (**F**eedback, **R**esponsibility, **A**dvice, **M**enu of options, **E**mpathy, and **S**elf-efficacy) with a goal of motivating individuals to begin the drinking change process prior to initiating treatment. Rates of treatment entry were 10% higher for the leaflet than the no-leaflet group, although the effect was not statistically significant (x = 3.61, p = 0.057). This study is the only one included in this review to specifically investigate the impact of a low-intensity intervention on treatment entry.

Wild and colleagues [39] studied six-month outcomes in a sample of current drinkers (N = 1722) randomly assigned to either brief personalized feedback on drinking norms or delayed-treatment. The brief personalized feedback intervention, presented as a mailed pamphlet, invited drinkers to compare their alcohol consumption with that of men or women in the general population. Although there was no main effect of experimental condition on drinks per drinking day for the entire sample, individuals who drank hazardous amounts (>14 drinks per week for men or >9 for women) improved more than those who drank less heavily; that is, the hazardous drinking × intervention effect was significant (B = −0.124, t = 2.5, p < 0.01). Thus, the intervention impacted individuals who were drinking in a hazardous fashion more than those who were nonhazardous drinkers.

Kavanagh and Connolly [40] evaluated the impact of a mail intervention on 204 men and women with an alcohol use disorder (abuse or dependence). The intervention was a single-blind randomized trial with a cross-over design wherein participants receiving the intervention either immediately or delayed by three months. The intervention was cognitive behavioral in nature and involved motivation enhancement, challenging overly positive alcohol expectancies, specifying drink refusal skills, and maintaining nonalcohol-related social support. The intervention was divided into eight components delivered weekly for the first month and biweekly thereafter. Compared with participants in the delayed condition, those in the active condition had a more significant reduction in alcohol consumption (Wald x^2^(1) = 7.46, p = 0.006). Participants cut their drinking almost in half but continued to drink at fairly high levels, with men reporting 27 drinks per week and women 14 drinks per week. Even so, the reduction in drinking is notable given that the average AUDIT score at intake was 22.3, indicating moderate alcohol dependence.

### Telephone interventions

Telephone interventions increase accessibility of care by eliminating the need for travel to a treatment center [41]. We identified two telephone studies that met inclusion criteria. One study randomized primary-care patients who screened positive for an AUD (alcohol abuse or dependence) to receive either a brief telephone motivational interviewing (MI) intervention (up to six sessions) or a four-page pamphlet on healthy lifestyles [31]. At three-month follow-up, men in the telephone condition experienced greater reductions in risky drinking days (30%) than men in the pamphlet (8%) condition (n = 201, p < 0.001); women experienced significant reductions in drinking in both the telephone (17%) and pamphlet (12%) conditions (n = 251; not significant). Participants who met diagnostic criteria for alcohol dependence improved as much as those who only met criteria for alcohol abuse.

A second telephone study [42] randomized emergency-department patients within five days of admission to a two-session telephone intervention or standard care (assessment only). Participants included individuals who screened positive for hazardous alcohol use (≥14 drinks per week for men or ≥7 for women, or ≥5 drinks per occasion for men or ≥4 per occasion for women). Both groups improved in drinking outcomes at three-month follow-up, but only the telephone group (mean change = −1.4; 95% CI, -3.0 to 0.2) reported significantly reduced impaired driving compared with the standard-care group (mean change = 1.0; 95% CI, -0.9 to 2.9) (p = 0.04). Similar to results of the bibliotherapy studies [36,39], individuals reporting heavier drinking experienced the most benefit from the telephone intervention.

### Computer-based interventions

The benefits of computer-based interventions include the potential for remote access, the ability for individuals to choose content they prefer, and increased appeal of multimedia applications. Hester and coworkers [43] evaluated the “Drinker’s Check-up,” a brief computer-based MI intervention that includes assessment, personalized feedback, and a decision-making module that takes about 45–60 minutes to complete. Individuals were solicited by media announcements and needed to be drinking in a problematic fashion as indicated by an AUDIT score >8 (the mean AUDIT score was 20 for all participants). Subjects were randomized to the intervention or a four-week waitlist (control condition). At one-month follow-up, individuals randomized to the intervention condition reported significantly lower levels of drinking from baseline to eight weeks (F (6,43) = 2.667; p = 0.027). At one-year follow-up, both the intervention and delayed intervention groups reported a 50% decline in quantity and frequency of drinking, indicating strong support for the intervention. At 12-month follow-up, it was also found that 28 of the 61 participants subsequently engaged in formal treatment or had attended AA. This help-seeking may have been inspired by content within the computer program, which included treatment referral information. These findings seem to support the apparent effectiveness of the intervention on subsequent help seeking, despite the lack of a no-intervention control.

Another study [32] evaluated a brief (15–20 minute) computer-based MI intervention for emergency-department patients. The study randomized 1139 individuals with AUDIT scores >5 to intervention or control conditions. The intervention consisted of computer-generated feedback about current drinking delivered on the computer and in a letter provided to the participant prior to leaving the emergency department. In addition to feedback on safe-drinking norms, the letter provided a FRAMES-based intervention [44]. Participants began the study at similar levels of hazardous drinking; however, at six months, 21.7% of the intervention group versus 30.4% of the control group met criteria for hazardous drinking (p = 0.008), and at 12 months, alcohol intake in the intervention group decreased by 22.8% compared with 10.9% in the control group (p = 0.02).

A third study [30] evaluated a brief (20-minute) computer feedback intervention known as “DrinkTest” for men recruited from a nationally representative panel in the Netherlands. Participants met Dutch criteria for hazardous alcohol use (≥15 drinks per week or ≥4 on a single occasion at least once per week) and were randomized to the intervention or to a control group that received an educational pamphlet. The intervention provided normative feedback on drinking, enumerated potential consequences of heavy drinking, and provided suggestions for reducing alcohol intake. At one-month follow-up, the intervention produced significant benefits, with 42% of those in the experimental condition reporting drinking within recommended limits compared with 31% in the control condition (x^2 =^ 6.67, p = 0.01). At six-month follow-up, the intervention effects were less strong, with drinking within recommended limits reported by 46% and 37% of the intervention and control conditions, respectively (x^2 =^ 3.25, p = 0.07).

### Internet-based interventions

The Internet offers another method for reaching individuals with AUDs. One form of Internet-based intervention involves assisting individuals in assessing and evaluating their own drinking. Cunningham and colleagues [45] tested the Internet-based “Check Your Drinking” intervention in a random sample of drinkers who met criteria for hazardous alcohol use (score of ≥4 on the three-item AUDIT-C). Participants were randomly assigned to either the “Check Your Drinking” intervention, which provided brief personalized normative feedback (approximately 10 minutes), or to a no-feedback control condition. Individuals scoring >11 on the AUDIT-C were categorized as problem drinkers. Problem drinkers assigned to the feedback condition reported a significant reduction in drinking at three-month follow-up (p < 0.05) and an additional reduction at six-month follow-up (p < 0.05), whereas problem drinkers in the control condition did not show significant reductions in drinking.

A study by Pemberton and colleagues [46] compared the effectiveness of two web-based alcohol interventions, “Alcohol Savvy” and “Drinker’s Check-up,” which were adapted for use within a military population. “Alcohol Savvy” is an alcohol-misuse prevention program that uses education about the risks of drinking and the benefits of moderate alcohol consumption to create motivation for individuals to make better decisions regarding alcohol use. The version of “Drinker’s Check-up” used this study was an online version of the intervention described previously in the computer section (MI for high-risk drinkers). Study participants (N = 3070) were active-duty military personnel who were voluntarily recruited through a variety of means at eight different installations. Participants did not need to meet any screening criteria for hazardous alcohol use. Randomization to either of the active conditions or a waitlist (control) condition was quasi-randomized; participants who lacked access to high-speed Internet were assigned to “Drinker’s Check-up” as their active condition due to the technical requirements of the “Alcohol Savvy” intervention. At one-month follow-up, participants who completed the “Drinker’s Check-up” reported significant reductions in average number of drinks per occasion (p > 0.05) compared with controls. The comparison between “Alcohol Savvy" and the control group was not significant.

Complex web-based alcohol treatment involves more elaborate forms of engagement beyond feedback interventions, such as communication between users, communication with clinical personnel, and/or content meant to be perused over weeks or months. The web-based “Drinking Less” intervention [47] includes interactive materials based on cognitive-behavioral and self-control principles and a moderated peer-to-peer discussion forum. In a study to assess its effectiveness, participants were block-randomized to either the intervention or a control condition involving a web-based psychoeducational brochure about the negative impacts of unhealthy alcohol use. Problem drinkers in the study included men who consumed ≥14 drinks per week or ≥4 drinks in one day, and women who consumed ≥10 drinks per week or ≥3 drinks in one day. Among individuals in the intervention condition, 17% decreased drinking to safe levels at six-month follow-up compared with only 5% in the control condition (OR = 3.66; CI 1.3-10.8; p = 0.006). Individuals in the intervention condition also reported an average reduction of weekly drinking of 11 drinks at six-month follow-up, compared with the control group’s reduction of only two drinks (OR = 5.86; CI, 5.86-18.10; p = 0.001). The vast majority (88%) of users of the website had never entered professional treatment, indicating that interventions of this nature may appeal to problem drinkers uninterested in traditional alcohol-treatment services.

An e-therapy alcohol intervention [48] included assessment, goal-setting, and regular interaction with counselors via email for up to three months. Participants (N = 156) who met the study’s criterion for problem drinking (≥15 drinks per week for men and ≥11 for women) were recruited via online advertising and randomized to three months of access to the website or to a waitlist (control condition). The intervention was facilitated by email contact from a counselor and occurred in two stages: a motivational stage that involved assessment of drinking consequences and feedback and a second phase that involved completing modules based on cognitive behavioral therapy for alcohol problems. At three-month follow-up, participants randomized to the intervention reduced their weekly alcohol intake by a 26 drinks compared with those in the control group, who decreased weekly alcohol intake by only two drinks (mean difference 95% CI, 15.69-35.80; p < 0.001). Almost 80% of participants met the *Diagnostic and Statistical Manual of Mental Disorders, 4*^*th*^*revision* (DSM-IV) criteria for mild alcohol dependence, and 76% reported never having received treatment for their alcohol problems.

Another intervention study of problem drinkers, conducted by Blankers and colleagues [49], evaluated the effectiveness of an Internet-based alcohol intervention (therapy alcohol online [TAO]), and Internet-based self-help (self-help alcohol online [SAO]). Participants scored >8 on the AUDIT (mean score for all participants, 19.5) and reported drinking >14 drinks per week on average. At total of 205 participants were randomized to TAO, SAO, or a waitlist (control condition). The SAO condition was a stand alone, fully automated, self-guided intervention based on cognitive-behavioral therapy and MI techniques. Participants in the SAO group also received support from other SAO participants in the form of an Internet-based forum. The TAO condition was a synchronous online intervention based on the same SAO treatment protocol but also included 40-minute text-based therapy chat sessions. Contact between TAO participants and participants could occur synchronously during chat sessions or asynchronously via email. At three-month follow-up, generalized regression models indicated significantly lower alcohol consumption for the TAO group (p = 0.002) and the SAO group (p = 0.03) relative to the waitlist group. From baseline to three-month follow-up, the TAO group reduced their weekly alcohol consumption by an average of 24 drinks compared with a reduction of 16 drinks in the SAO group and 12 in the waitlist group. The mean reduction in weekly drinking between the TAO and SAO groups was not significantly different at three-month follow-up, but a significant difference favored the SAO group (p = 0.03) at six months.

A study of the “Down Your Drink” web-based intervention [50] compared an interactive website employing elements of MI with cognitive behavioral techniques with a more static, text-based version of the site that focused on harms caused by excessive alcohol consumption. Interactive components of the “Down Your Drink” website were divided into three stages focusing on individual responsibility for change, deciding on change, and maintenance of change, and included e-tools such as a “thinking drinking diary” in which users could record their alcohol consumption along with emotional and behavioral triggers. Hazardous drinkers who had AUDIT-C scores >5 (N = 7935) were randomized to either the enhanced “Down Your Drink” website or to the text-based site (control condition). At three-month follow-up, the intervention group reported a substantial reduction in alcohol consumption (46.3 to 26.4 drinks per week) as did the control group (45.7 to 25.6), but the difference between groups was not significant (OR, 1.03; 95% CI, 0.97-1.10.) Changes were maintained in both groups at 12 months.

The “Drinking Less” intervention described earlier was also tested via a television-supported platform [51]. Problem drinkers (N = 181) were recruited using the “Drinking Less” website and assigned to receive weekly DVDs (five in total, 25 minutes each) and a self-help manual or to a waitlist (control condition). The content of the DVDs paralleled that of the “Drinking Less” website but also showed two problem drinkers who underwent and completed the intervention with a trained addiction coach. At five-week follow-up, 40% of individuals in the intervention condition were engaging in low-risk drinking compared with only 7% of individuals in the waitlist condition (x^2^(1) = 28.3; p = 0.001; OR, 9.4; 95% CI, 3.7-23.9). The reductions in drinking remained at three-month follow-up.

## Discussion

A variety of low-intensity interventions can engage individuals and effectively reduce drinking. Moreover, these strategies offer easier access and flexibility to individuals who misuse alcohol and circumvent some of the barriers to entry into traditional treatment. They also offer the potential for greater privacy, although strong encryption and other safeguards are needed to ensure that individuals’ data remain private and confidential for online interventions. In the following section, we summarize three key findings from existing studies of low-intensity interventions and identify three important areas for future research on stepped-care intervention models.

### Key findings about low-intensity interventions

*Low-intensity interventions attract individuals who have not previously sought help.* Several of the studies discussed herein included participants who had never sought treatment previously or who expressed disinterest in formal treatment options but accepted the low-intensity intervention. Mail [35], Internet-based [47,48], and bibliotherapeutic [34] interventions can attract individuals who have never previously sought treatment, including those who meet criteria for alcohol abuse or dependence [34,35]. None of the other studies reviewed above reported on participants’ prior help-seeking history, so it is not possible to determine whether any one type of intervention is especially attractive to individuals with AUDs. Moreover, we are unaware of studies that have offered a low-intensity intervention or any treatment to individuals who report disinterest in formal or informal treatment options. More comparative information is needed about which low-intensity interventions are most attractive to individuals with AUDs who are unlikely to attend more traditional formal treatment. We also need to know whether some modalities are more popular than others for specific groups of individuals. In this vein, less educated individuals were more likely to be drawn to the “Drinking Less” intervention when it was delivered via television [51] than when it was delivered via the Internet [47]. Another important question for future studies is whether low-intensity interventions, offered in the context of a stepped-care intervention, are more likely to attract problem drinkers relative to offering more intensive treatment.

*Low-intensity interventions can benefit individuals with more severe AUDs.* Meta-analytic studies suggest that brief interventions conducted in settings such as primary care tend to be more effective for individuals with less severe AUDs [23]. Similar to face-to-face brief interventions, all of the low-intensity interventions reviewed herein significantly reduced alcohol use among participants. However, several of the studies also reported significantly reduced alcohol use among patients with low to moderate alcohol dependence. For example, Brown and colleagues [31] found that a telephone-based intervention produced equivalent outcomes for participants who met criteria for abuse or dependence. A bibliotherapeutic intervention reduced drinking among participants meeting criteria for alcohol abuse or dependence by 50% [40], another bibliotherapy intervention reduced alcohol use by 30% [35], and two online interventions reduced drinking by 50% [47-49]—despite the fact that participants in these four studies reported average AUDIT scores of >19. Thus, low-intensity interventions appear to significantly reduce drinking among hazardous alcohol users and can also engage and reduce drinking among individuals with more severe alcohol-related problems.

*Low-intensity interventions may lead to subsequent help-seeking.* Low-intensity interventions may inspire a self-change attempt which, if unsuccessful, leads to subsequent help-seeking [35]. Almost half of the individuals who participated in the computer-based “Drinker’s Check-up” sought some form of additional help within the next 12 months [43]. Almost one-quarter of the individuals in the mail-based intervention [35] sought some form of help for their alcohol use in the subsequent year, including “control” participants, potentially inspired by completion of study assessments. A number of studies reviewed by Apodaca and colleagues [34] indicated that individuals who had previously not entered formal treatment or mutual-help groups did so after participating in bibliotherapy. A majority of individuals who engaged in e-therapy indicated that they would consider seeking treatment, and some decided to participate in face-to-face therapy [48]. Thus, consistent with prior findings showing that unsuccessful quit attempts often precede help-seeking [19-21], low-intensity interventions may help individuals engage in subsequent treatment even when the interventions themselves are unsuccessful at precipitating change. It is not possible to establish definitively the effects of these interventions on help-seeking, as participants in the intervention and control conditions either sought subsequent help at equal rates, subsequently received the same intervention, or results were not reported separately for the different groups.

Assessing subsequent help-seeking in the context and aftermath of low-intensity interventions could provide valuable data to inform stepped-care interventions. We need to know whether individuals seek help after a low-intensity intervention because they resumed problematic drinking (failed to meet goals) and/or whether low-intensity interventions help dispel concerns about treatment and thereby increase individuals’ motivation to enter into it.

### Key questions for implementing stepped-care interventions

*How can low-intensity interventions be structured to initiate a stepped-care intervention?* In general, low-intensity interventions appear well-suited as the first step of a stepped-care intervention [35,47,48]. However, a number of important questions need to be addressed regarding how best to integrate low-intensity interventions into standard treatment. For example, should individuals who participate in a low-intensity intervention but are unsuccessful at reducing their alcohol use be actively referred to more intensive treatment, or should they just be provided with referral information? Is it more appealing for a low-intensity intervention to be remotely accessible and independent than for it to be specifically tied to an alcohol treatment center? Stepped-care models are a potentially efficient method for titrating care for individuals with AUDs, but attention should also be directed to whether or not any given model successfully increases the reach of alcohol treatment.

Another potential implementation pathway for low-intensity interventions is as population-based interventions that focus more on the overall impact on groups of individuals than on efficacy for each individual. Within this framework, the “impact” of an intervention is not only its efficacy, such as a 5% decrease in hazardous alcohol use, but the efficacy multiplied by the number of participants [52]. The efficacy of online interventions such as “Drinking Less” in the Netherlands, which decreased the rate of hazardous alcohol consumption by 17%, may appear modest compared with most treatment outcome studies that report abstinence rates of 30-40% [53]. However, with the ability to reach a large number of homes, the "Drinking Less" website could have a substantial population-based impact on drinking.

In countries such as the US, where higher intensity treatment options are widely available, a variety of low-intensity interventions can be included as part of population-based stepped intervention, as currently exists with “AlcoholScreening.org” [54]. Similarly, the computer-based “Drinker’s Check Up” [43], which combined a low-intensity intervention with referral information, resulted in 45% of the participants seeking additional help. Combining low-intensity intervention with referral to intensive treatment in the US is feasible given the existence of online resources such as the Substance Abuse Treatment Facility Locator [55] and mutual-help meeting locators for groups such as AA [56], Smart Recovery [57], and Life Ring [58].

The success of smoking quitlines offers another model for population-based alcohol interventions [59]. Smoking quitlines have strong empirical support [60] and additional advantages such as convenience, relative anonymity, and ease of creating a structured counseling protocol. Lichtenstein and colleagues [59] identify problem drinking as well-suited to the quitline model, given that the disorder is highly prevalent, that suitable protocols for intervention currently exist, and that the widespread negative impacts of hazardous drinking provide governments with a stake in funding such an enterprise. Alcohol quitlines could follow problem drinkers long enough to determine the success or failure of the intervention, and then, per the stepped care model, offer referral to more intensive treatment to individuals who remain motivated to reduce their level of drinking.

*What are the mechanisms that underlie the benefits of low-intensity interventions?* Gaining a better understanding of how low-intensity interventions work, particularly within the process of seeking help, can inform the creation and implementation of stepped-care interventions. We know that alcohol-related problems, particularly multiple problems, predict help-seeking and reductions in drinking. All of the low-intensity interventions in this review provided online or telephone feedback about negative consequences associated with drinking [31,48], automated initial assessments [45], or self-help materials [43,47]. With one exception [35], all of the studies found better alcohol outcomes when personalized feedback and informational advice were provided than when they were not provided. However, to more fully understand the impact of personalized feedback on subsequent drinking, we need to examine the extent to which the feedback changes the perceived severity of alcohol problems or another potential mediator, and whether any such changes are tied to changes in drinking [61]. Support for such a causal chain could substantiate the effectiveness of personalized feedback and contribute to a better understanding of “why” or “how” low-intensity interventions work.

We also need to examine whether certain aspects of low-intensity interventions, such as feedback about negative consequences of drinking or providing information about normative drinking, are especially likely to lead to subsequent treatment seeking. Is an initial assessment and evaluation sufficient to motivate help-seeking, or is participation in the intervention and an unsuccessful quit attempt the key to help-seeking? Gaining a better understanding of how low-intensity interventions help individuals recognize the severity of their alcohol-related problems offers the hope of telescoping the normal help-seeking process and thereby averting a considerable degree of alcohol-related harm.

*Are some low-intensity interventions more beneficial for some groups (e.g., men or women) than others, and are there additional low-intensity interventions that should be considered?* Design of stepped-care interventions should take into account the potential for low-intensity interventions to be more or less effective for some groups. More information is needed about the extent to which low-intensity interventions are effective across diverse population groups. As noted, Brown and colleagues [31] found that a telephone-based intervention was more effective than a pamphlet-only condition for men but not for women. More generally, there is a need to examine whether low-intensity treatment interventions are differentially effective across gender and sociodemographic and racial/ethnic groups. For example, less well-educated individuals were more likely to be drawn to the “Drinking Less” intervention when it was delivered via television [51] than when it was delivered via the Internet [47]. It may be appropriate to adopt theory-based, data-driven cultural adaptation techniques [62] to modify low-intensity interventions for different cultural groups. In addition, given that almost 20% of the US population speaks a language other than English [63], future research should develop and test low-intensity interventions for AUDs in languages other than English.

A recent report by the Pew Research Center’s Internet and American Life Project [64] indicates that 83% of American adults own cell phones, and 73% send and receive text messages. Most studies evaluating the impact of text messages on health care focus on health activities such as appointment reminders, but a growing number deliver health-behavior change messages. A recent review found positive health benefits from SMS-delivered messages targeting diabetes self-management and smoking cessation [65]. Thus far, only college students have been the focus of text-message studies targeting alcohol use [66]. However, with individuals aged 30–49 sending or receiving an average 27 of messages per day, text-message interventions should be considered as a potential low-intensity intervention targeting adult alcohol use. In addition to sending alcohol-related health messages, an online text system could record drinking goals, ask users how well such goals are being met, and direct those not meeting drinking goals to websites providing treatment and mutual-help resources.

## Conclusion

Rather than developing new forms of intensive treatments for AUDs when current treatments work reasonably well, more effort should focus on expanding the reach of treatment by developing more accessible interventions and exploring how to integrate them into existing alcohol treatment systems. Low-intensity interventions are flexible and can be tailored to address many of the perceived barriers that hinder individuals with AUDs from obtaining timely help. Substantial evidence indicates that low-intensity interventions can engage individuals who shun current treatment options, reduce problematic alcohol use, and may even motivate individuals who need it to engage in more intensive treatment. Given the existence of effective low- and higher intensity interventions to address AUDs and the low levels of treatment uptake, greater attention needs to be focused on implementation-oriented aspects of stepped-care interventions. Several issues regarding the implementation of stepped-care interventions still need to be addressed by the literature, such as identifying the best structure for stepped-care models, understanding the impact of stepped-care interventions on motivation for changing alcohol use, and comparing the effectiveness of stepped-care models across diverse populations.

## Competing interests

The authors declare that they have no competing interests.

## Authors’ contributions

JM and RM collaborated on the conception of the manuscript. JM wrote most of the manuscript with the exception of the Methods section and table that was written by JA. JA carried out all literature reviews for the paper. RM provided editorial feedback. All authors read and approved the final manuscript.
